# Mesenchymal Hamartoma in Children: A Diagnostic Challenge

**DOI:** 10.1155/2019/4132842

**Published:** 2019-09-16

**Authors:** Muhammad Rehan Khan, Larry A. Binkovitz, Thomas C. Smyrk, D. Dean Potter, Katryn N. Furuya

**Affiliations:** ^1^Division of Pediatric Gastroenterology & Hepatology, Department of Pediatrics, Mayo Clinic, Rochester, Minnesota, USA; ^2^Department of Radiology, Mayo Clinic, Rochester, Minnesota, USA; ^3^Department of Pathology, Mayo Clinic, Rochester, Minnesota, USA; ^4^Division of Pediatric Surgery, Department of Surgery, Mayo Clinic, Rochester, Minnesota, USA

## Abstract

Mesenchymal hamartoma is a benign tumor of the liver with a poorly understood pathogenesis. It is uncommon in older children, especially after 2 years of age. The signs and symptoms may be nonspecific; therefore, a high index of suspicion is required for diagnosis and treatment. We report a 5-year-old previously healthy male who presented with acute abdominal pain, fatigue, and fever. He was diagnosed with pneumonia initially and treated with antibiotics. A computed tomography (CT) scan done for evaluation of his persistent abdominal pain demonstrated a hepatic mass. Follow-up magnetic resonance imaging (MRI) of the liver demonstrated multiple serpiginous tubular-type structures, read as possible Caroli syndrome. He had a normal abdominal examination and normal biochemistries including alanine aminotransferase, aspartate aminotransferase, gamma-glutamyl transferase, alkaline phosphatase, and alpha-fetoprotein. He was referred to our institution for second opinion. On further review of his imaging studies, the lesion was thought to be a mesenchymal hamartoma. He subsequently underwent resection of the mass. Pathology confirmed the diagnosis of mesenchymal hamartoma.

## 1. Introduction

Mesenchymal hamartoma (MH) is a benign tumor of the liver. It is the second most common benign pediatric hepatic tumor after infantile hemangioma and usually derives from mesenchymal tissue components [1]. More than two-thirds of the cases are seen in children less than 2 years of age. The incidence of mesenchymal hamartoma is extremely rare in older children and adults [2, 3]. Most of these tumors are located in the right lobe of the liver although some cases have been reported in the left lobe as well [4, 5]. The diagnosis of this tumor is difficult because of nonspecific clinical symptoms and lack of definitive laboratory studies. Radiological imaging is crucial for diagnosis.

## 2. Case

A 5-year-old previously healthy male was referred to our institution for further evaluation of a liver mass. By history, he developed fatigue after participation in a fishing tournament 4 weeks prior to presentation followed by abdominal pain the next day. He was taken to the local hospital for evaluation of his symptoms where he was found to have a fever of 38.9°C. His evaluation included routine laboratory testing and chest X-ray. Based on chest X-ray findings and his symptoms, he was diagnosed with pneumonia and was treated with antibiotics. His abdominal pain at that time was thought to be referred pain. He completed a course of antibiotics but continued to have mild abdominal pain. A CT scan of the abdomen was done for further evaluation of his persistent abdominal pain, and it showed a multicystic-appearing lesion within the inferior aspect of the right hepatic lobe. Subsequently, a magnetic resonance imaging (MRI) exam of the abdomen was performed to further evaluate the hepatic mass. The MRI showed multiple serpiginous tubular-type structures predominantly within segment 5 with questionable peripherally located foci of T2 signal hyperintensity seen within the structures. These findings were thought to be consistent with possible focal Caroli syndrome. At that time, laboratory evaluation showed normal liver function tests with a prothrombin time (PT) of 11.6 sec; international normalization ratio (INR) 1.1; fibrinogen 435 mg/dL; alanine aminotransferase (ALT) 21 U/L; aspartate aminotransferase (AST) 26 U/L; and gamma-glutamyl transferase (GGT) 10 U/L. His inflammatory markers were slightly elevated with CRP 5.4 and ESR 30. His alpha-fetoprotein (AFP) was normal (0.8 IU/mL). Antibodies to *Entamoeba histolytica* and *Echinococcus* were checked and were negative. Because of persistent abdominal pain and no clear diagnosis of his liver mass, the patient was then referred to our institution.

**Figure 1:**
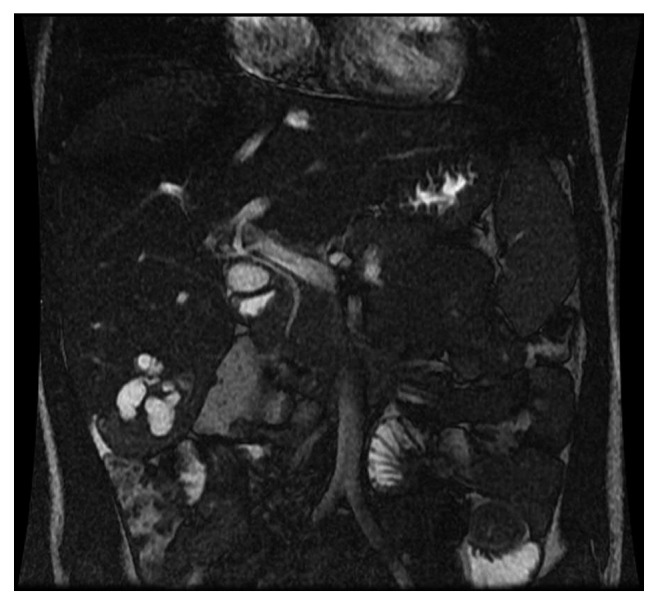
Clusters of multiple T2-bright circumscribed cystic lesions in segment 6 measuring <2 cm in greatest dimension suggestive of mesenchymal hamartoma.

**Figure 2:**
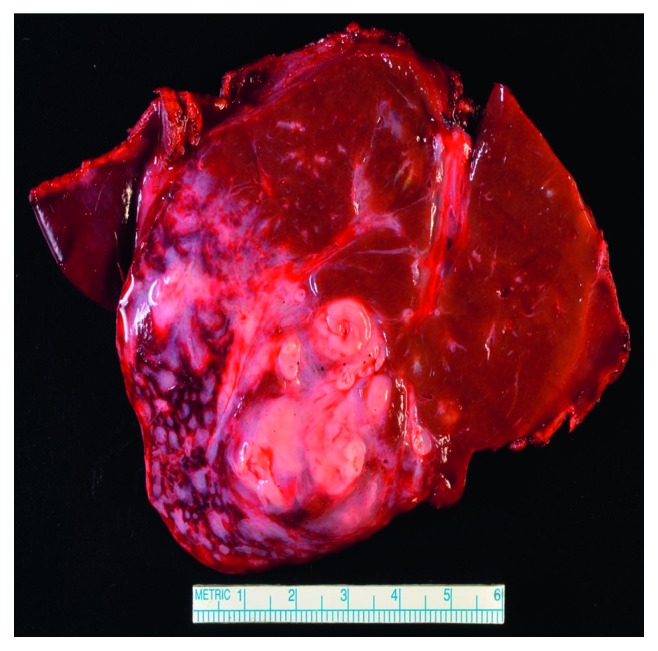
Gross specimen, resected tumor.

**Figure 3:**
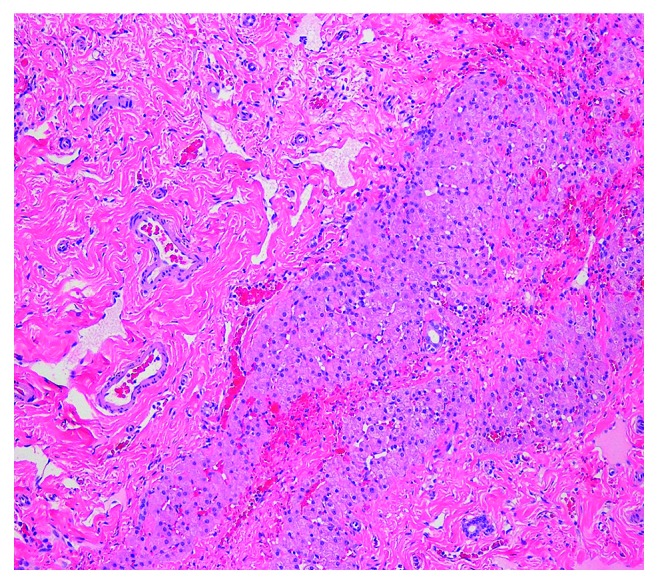
Puzzle-shaped pieces of hepatic parenchyma embedded in hyalinized matrix (hematoxylin and eosin stain with 200x magnification).

The review of the MRI at our institution showed clusters of multiple T2-bright circumscribed cystic lesions in segment 6 measuring <2 cm in greatest dimension which was suggestive of mesenchymal hamartoma (Figure 1). Pediatric surgery was consulted, and he subsequently underwent a hepatic lobectomy with resection of the ∼6 × 12 cm mass (Figure 2). The pathological review of the resected mass showed puzzle-shaped pieces of hepatic parenchyma embedded in hyalinized matrix (hematoxylin and eosin stain with 200x magnification), consistent with a mesenchymal hamartoma (Figure 3). The patient did well postoperatively and was asymptomatic at the time of his follow-up visit 1 month later.

## 3. Discussion

Benign tumors of the liver are relatively rare in children. Mesenchymal hamartoma was first described in the literature by Edmondson in 1956 [6]. Prior to this publication, there was no consensus regarding its specific histologic pattern, and it was described by various names in the literature such as giant-cell lymphangioma, bile-cell fibroadenoma, cavernous lymphangiomatoid tumor, and pseudocystic mesenchymal tumor [2]. Although the exact etiology is not clear, mesenchymal hamartomas seem to originate from a congenital, localized abnormality in ductal plate development [7]. It is typically composed of loose mesenchyme with variably sized cysts lacking an epithelial lining, accompanied by islands or cords of hepatocytes admixed with bile ducts and blood vessels [8].

The diagnosis of mesenchymal hamartoma still remains a challenge. Children can present with nonspecific symptoms such as abdominal pain, fatigue, and fever. Laboratory findings may also be normal. In some cases, an elevated alpha-fetoprotein may be seen [9]. The diagnosis of these tumors is often delayed until they are big enough to cause abdominal distension or a compression effect on surrounding structures [2]. However, with advances in imaging, early diagnosis is possible if the index of suspicion is high. Advanced imaging is usually helpful in the early diagnosis although nonspecific findings may also be noted. A delay in diagnosis can lead to complications from local compression [10]. Rarely, malignant transformation has also been reported [11].

Differentiating between mesenchymal hamartoma and other tumors such as an undifferentiated embryonal sarcoma is difficult based on imaging alone [12]. The precise diagnosis of mesenchymal hamartoma relies on histological evaluation of the tissue. However, because of the risk of recurrence and malignant transformation, the gold standard for the diagnosis and treatment of mesenchymal hamartoma is complete surgical excision [13]. The role of liver transplant is not clearly defined in this benign condition. However, if the tumor is unresectable or recurs after partial hepatectomy, liver transplantation remains a viable therapeutic option in both children and adults [9, 10].

## 4. Conclusion

Mesenchymal hamartoma may have nonspecific signs and symptoms. Advanced imaging and expertise in interpretation of imaging findings is necessary for the diagnosis as routine investigations may be normal. Complete surgical resection is the standard of care for both diagnosis and treatment.
